# How and when paternalistic leadership influences service innovative behaviour while inhibiting counterproductive work behaviour among healthcare professionals: the roles of perceived supervisor support and public service motivation

**DOI:** 10.1108/JHOM-08-2024-0333

**Published:** 2025-01-30

**Authors:** Muzammil Hussain, Trong Tuan Luu, Timothy Marjoribanks

**Affiliations:** Swinburne University of Technology, Hawthorn, Australia

**Keywords:** Social exchange theory, Paternalistic leadership, Service innovative behaviour, Counterproductive work behaviour, Public service motivation, Healthcare

## Abstract

**Purpose:**

Healthcare is a service industry where fulfilling the needs of patients (customers) is challenging. Various factors, including cost, system complexity, staffing behaviours and technological advances, play vital roles. Drawing upon social exchange theory, this study seeks to determine how paternalistic leadership (authoritarianism, benevolence and morality) influences employee service innovative behaviour and counterproductive work behaviour via perceived supervisor support in the healthcare sector. Additionally, the study investigates the role of the public service motivation of individuals as a moderating factor in this relationship.

**Design/methodology/approach:**

A pilot study and a main study were conducted to test the hypotheses. We collected data from healthcare professionals in Pakistan’s large public, private and semi-government hospitals. We applied bootstrapping with 5,000 replications and structural equation modelling to analyse the data.

**Findings:**

Results indicate that authoritarianism was negatively associated with service innovative behaviour, whereas benevolent and moral behaviours were positively associated with service innovative behaviour via perceived supervisor support (mediation). Our findings shed light on the moderating role of public service motivation.

**Originality/value:**

This empirical quantitative study has several theoretical and practical implications. Findings of our study provide evidence that a paternalistic leadership style can influence both positive (service innovative behaviour) and negative (counterproductive working behaviour) working behaviours simultaneously via perceived supervisor support at an individual level in the service (healthcare) industry. This study also highlights the moderating role of public service motivation as an individual motivation factor.

## Introduction

Research on employee service behaviours is gaining scholarly attention because the service sector, including healthcare, struggles to deliver services effectively in today’s dynamic environment (Gautam *et al*., 2022; Hofmeister *et al*., 2024). Employees demonstrate various service behaviours at work, which can be divided into two broad categories: productive and counterproductive (Sypniewska, 2020). One form of productive working behaviour is service innovative behaviour (SIB). SIB is the generation, promotion, and execution of novel and useful ideas by employees to enhance customer service. SIB also includes the extent to which employees develop new and useful ideas, spread ideas to colleagues, and implement those ideas or help others to implement those ideas (Janssen, 2000).

Creativity and innovation have moved from “good to have” to “must have” for modern organisations seeking to achieve a competitive advantage and sustain corporate growth (Wang *et al*., 2019; Tajeddini *et al.*, 2020). Moreover, it is becoming essential for service-centred organisations to offer variety and innovation to create value in services (Akter *et al*., 2022; Rasheed *et al*., 2024; Xie *et al*., 2024). Similarly, innovation in procedures may help to improve healthcare service quality, effectively respond to patient’s service demands, and increase patient satisfaction (Weintraub and McKee, 2019; Papa *et al*., 2020; Xie *et al*., 2024). Healthcare organisations rely in part on their employees to serve patients and improve service quality, as service innovation is accomplished importantly at the individual level (Tajeddini *et al.*, 2020). Healthcare employees may engage in SIB in various technological, integrative and service-based aspects (Guarcello *et al*., 2020).

At the same time, healthcare managers face challenges regarding employees' negative or counterproductive working behaviours, which have been reported as the darker side of employees' extra-role work behaviours (Zhao *et al*., 2022; Atiku *et al*., 2023). Employee counterproductive working behaviour (CWB) is a form of negative working behaviour which can be expressed in various forms, such as aggression, unethical behaviour, delinquency, workplace deviance, retaliation, sabotage, violence, emotional abuse, bullying, misconduct, damage, and property theft (Fida *et al*., 2015; Robinson and Bennett, 1995). CWBs have become a critical issue in organisations globally (Zhao *et al*., 2022).

A growing body of research is integrating leadership behaviours to influence SIB and CWB. However, there is a need to explore this further, particularly in Eastern societies with collectivist and high-power distance cultural values where leaders are expected to exhibit different behaviours than leaders in Western societies where individualistic cultural values predominate (Bedi, 2020). A leadership style known as Paternalistic Leadership (PL) (authoritarianism, benevolence, and morality) has gained growing interest among researchers (Bai and Wendy Pan, 2022; Bedi, 2020; Hiller *et al*., 2019; Chan, 2024; Demeke *et al*., 2024), as a potential means of fostering various employee behaviours in Eastern contexts. PL is “a [leadership] style that combines strong discipline and authority with parental benevolence and moral integrity” (Farh and Cheng, 2000, p. 84) to maintain role obligation and collective harmony among leaders and employees. Existing studies have shown that PL plays a dual role (authoritative and benevolence/morality) in influencing employee outcomes.

In this context, the present study engages with four gaps in existing knowledge. First, drawing upon social exchange theory (SET), the primary objective of the present study is to investigate how PL dimensions influence positive (SIB) and negative (CWB) forms of extra-role work behaviours in the healthcare sector. A few studies have examined associations between PL dimensions and SIB (Nazir *et al*., 2020; Karakitapoğlu-Aygün *et al*., 2019; Sürücü *et al*., 2024). Similarly, some PL studies have investigated CWB, such as authoritative leadership with CWB (Qi *et al*., 2020). SIB and CWB (positive and negative forms of employee extra-role behaviour), however, have rarely been investigated simultaneously in a PL study. The current study aims to bridge this gap.

Second, despite evidence existing on the association of PL with various employee behaviours (Bedi, 2020; Khorakian *et al*., 2021; He *et al*., 2021), the mechanisms underlying the relationship between PL dimensions and employee behaviours (SIB and CWB) are not fully understood. Prior studies on PL and employee behaviour have focused on different mediating factors such as self-efficacy (Wang *et al*., 2022), job-embeddedness (Khorakian *et al*., 2021), work-family conflict (He *et al*., 2021), person-organisational fit (Grobler and Grobler, 2021), organisational cynicism and work alienation (Jiang *et al*., 2019). These studies have neglected, however, the role of leaders' support despite its significance in promoting service innovation (Rasheed *et al*., 2024) and reducing counterproductive work behaviours (Guo *et al*., 2023). The current study attempts to fill this gap by positing that perceived support from the supervisor (PSS) anticipates a mediation effect between PL dimensions and employee service behaviours (SIB and CWB) among healthcare workers.

Third, this study proposes that public service motivation (PSM), that is, “an individual’s orientation to delivering public services to do good for the community” (Perry and Wise, 1990), has the potential to moderate the relationships between PL dimensions and mediating variable (PSS) in the health care context. PSM is a conceptualisation of motivation to serve the public, including rational, normative and affective motives (Perry and Wise, 1990). Prior PL studies have focused on various moderating variables such as benevolent leadership, power differences (Wang *et al*., 2022), career stage (Khorakian *et al*., 2021), cross-cultural adaptability (Wang *et al*., 2022; He *et al*., 2021), and perceived HRM strength (Jia *et al*., 2020). However, few PL studies (Karolidis and Vouzas, 2019; Petersen, 2021; Potipiroon and Faerman, 2020) have investigated PSM as a moderating variable in the public sector. We seek to extend these insights in this paper, by focusing on the moderating role of PSM, where PL dimensions influence SIB and CWB in both public and private sectors.

Fourth, despite the importance of the dual role of PL for employee performance in the manufacturing (Opazo-Basáez *et al*., 2022) and services industries, such as tourism and hospitality industry (Khorakian *et al*., 2021; Hoang *et al*., 2023; Hussain *et al*., 2023), understanding of PL in initiating employee’s SIB along with CWB in the healthcare sector remains under-researched. This study aims to advance PL research by unpacking its impact on both SIB and CWB in the healthcare sector. Moreover, the current study responds to calls for future research into more intervening variables, contexts, and sectors (Bedi, 2020; Chaudhary *et al*., 2021; Chan, 2024; Singh and Vashist, 2024). The study does this by investigating the proposed conceptual model in a context (Pakistani healthcare) that is different from where many PL studies have been conducted, including China’s education sector (Jia *et al*., 2020; Qian and Walker, 2021), China’s manufacturing sector (Chan, 2024), the Taiwan high-tech sector (Huang and Lin, 2021) and the Korean construction sector (Chai *et al*., 2020). The Pakistani healthcare services system is complex due to its formal and informal subsystems, many of which are run by the federal and provincial governments. There are three major categories of hospitals in the formal healthcare system: public-sector, private-sector, semi-government (an institution run by the state government with private entity shareholders), and non-government organisations (NGOs) trust hospitals. The informal healthcare system includes home health workers (registered employees who visit homes to provide services such as vaccinations) and homoeopathic practitioners (registered traditional healers specialising in Arabic and Chinese medicine).

## Hypotheses development and theoretical framework

### Social exchange theory (SET)

SET, developed by Blau (1964), provides an overarching framework for the present study to explain the relationship between PL and employee behaviours (Chen *et al*., 2014). SET is based on the norms of reciprocity and negotiated relations to have win-win situations (Cropanzano and Mitchell, 2005). Within SET, fairness and social standards are expected to be expressed by each party to promote feelings of indebtedness and obligation under the exchange process. Social exchange is the exchange of activities on a daily basis, whether tangible or intangible, and whether rewarding or not, among at least two members of a society. For instance, paternalistic leaders should reflect employee reciprocity in terms of support, care, empathy, and value. In response, employees should exhibit gratitude and should perform their duties at or, preferably, above the expected level. Similarly, the benevolence and moral behaviour of leaders can trigger the social exchange process and induce constructive emotions towards organisations and supervisors. We propose, based on SET, that PL encourages exchange-related behaviours between leaders and followers in the workplace (Zhang *et al*., 2022).

### Authoritarian leadership (AL) and employee service behaviours

Paternalistic leadership (PL) is congruent with high power distance and collectivism. The first dimension of PL is authoritarian leadership (AL), which is referred to as “higher-ups govern” and “lower obey”. AL behaviour offers clear directions, determines rewards and punishments, and initiates structures (Jia *et al*., 2020), which help employees to focus on specific and unambiguous goals (Wang and Guan, 2018). The authoritarian leader directs authority and control over their subordinates, and they expect unquestioned obedience (Cheng *et al.*, 2004). There are two key dimensions to this relationship.

First, the relationships between authoritative leaders and employees are formal due to hierarchical differences, often short-term, based on an exchange of pay and benefits, which may weaken the sense of extra-role efforts among employees (Bedi, 2020; Khorakian *et al*., 2021). Furthermore, given social exchange norms, individuals are less likely to reciprocate AL behaviour to perform SIB. Jia *et al*. (2020) explained that when employees are confronted with threats of violation of rules and pressures, they demonstrate defensive behaviours and may restrict themselves to extra-role service behaviours to protect themselves from disciplinary actions.

Second, authoritarian behaviour is often demanding and pressuring. It may disempower followers and reduce their willingness and ability to generate novel and innovative ideas (Zhang *et al*., 2022). Moreover, leaders' strict and controlled behaviour may influence the opportunities to establish a creative working environment. For instance, AL controls the followers' autonomy and reduces followers' job satisfaction and organisation-based self-efficacy. Sometimes, authoritative leaders instruct employees to work without good support. Further, such leadership sometimes exhibits injustice because employees feel that they are instructed to perform tasks without sufficient resources. Without such support from authoritative leaders, employees are less likely to form a social exchange relationship with the leader, and thereby are also less likely to reciprocate the leader action and are less likely to engage in extra-role behaviours. Zhang *et al*. (2022) found that employees tend not to socially exchange with the authoritative leader to engage in innovative behaviour. Based on the social exchange norm, we hypothesise that the above-discussed conditions vary considerably from those that could enhance innovative behaviour. Thus, we hypothesise:

H1a. AL is negatively related to SIB.

Early social psychological studies revealed that leader behaviours that impose huge pressure on followers might increase spontaneous aggression and hostile behaviours among those followers over time (Lewin *et al*., 1939). First, authoritarian leaders with strict discipline make all critical decisions themselves (Hanaysha, 2023). Consequently, subordinates may adopt a defensive silence that affects their behaviours and leads to negative subordination. Healthcare workers face stressful situations at work (Luu and Djurkovic, 2019), and frustrated employees may express aggression towards colleagues or patients rather than the authoritative leader due to fear of the leader (Park *et al.*, 2022). Second, authoritarian leaders' strict and disciplined behaviour is sometimes perceived as less democratic (Yin, 2022). As a result, subordinates may attempt to adapt their service behaviours and job attitudes downwards (Pizzolitto *et al*., 2022), including counterproductive behaviours such as violation of ethical norms, unplanned absenteeism, aggression, and verbal or physical abuse with colleagues and patients. Viewed through the lens of SET, we hypothesise that:

H1b. AL is positively related to CWB

### Benevolent leadership (BL) and employee behaviours

The benevolence dimension of PL or benevolent leadership (BL) is referred to as the Chinese concept of *shi-en* (granting favours) (Farh and Cheng, 2000). It is stated to express individualised, comprehensive concern for employees and their families' wellbeing (Farh and Cheng, 2000). Benevolent leaders demonstrate compassion and promote productive behaviours, which help their team members feel better about themselves and help them to form stronger social bonds. Additionally, BL provides opportunities for employees to develop professionally and personally (Lee *et al*., 2023). These encouraging emotions and personal acts of kindness could deepen interpersonal ties and encourage reciprocity.

Moreover, the positive behaviour of leaders can promote social harmony and trust among employees towards leaders (Lin *et al*., 2019). For example, high-quality interactions among leaders and subordinates will tend to include sharing opinions more comprehensively, which will equip subordinates to enhance the information that leads to developing service innovation (Wu *et al*., 2023). When individuals have high-quality working relationships, they are likely to take risks in trying new ideas in procedures (Wang *et al*., 2019). In light of SET, when benevolent leaders treat employees with kindness and care, employees reciprocate by respecting their leaders, putting in extra effort to enhance productivity, and going beyond required job duties based on a sense of indebtedness (Blau, 1964). We hypothesise:

H2a. BL is positively related to SIB.

Through the lens of SET, when benevolent leaders express individualised care and support towards employees, employees reciprocate not only by extending responsibilities in the form of extra-role performances (He *et al*., 2021), but also by being less likely to engage in harmful behaviours at the workplace. For example, when benevolent leaders act like parental figures to show long-term care and concern for employees' job-related and personal wellbeing, employees develop warm feelings and gratitude toward the leader (Bai and Wendy Pan, 2022). This reciprocity norm leads to an emotional bond between leader and employees, and this reciprocal relationship continues the positive cycle in light of SET (Blau, 1964). The behaviour of benevolent leaders appears to be a positive and welcoming leadership style that predicts lower CWBs. We hypothesise:

H2b. BL is negatively related to CWB.

### Moral leadership (ML) behaviour and employee behaviours

ML refers to *shu-de* (setting an example) and expressing moral deeds, demonstrating spiritual integrity, respect, admiration, and collective good rather than self-interest (Farh and Cheng, 2000). Moral leaders value morality and express their beliefs by demonstrating spiritual integrity, respect, admiration, and a commitment to the collective good rather than self-interest (Hoang *et al*., 2023; Wang *et al*., 2019). Moral leaders are value-driven, encourage individual concerns for process, practice self-discipline, lack self-centredness (Farh and Cheng, 2000), and act as principled decision-makers focused on the greater good of employees and organisations (Khorakian *et al*., 2021). Leaders are likely to serve as moral role models and exert referent power on employees, promoting an emotional bond and contributing to staff’s commitment (Grobler and Grobler, 2021). Viewing from the perspective of SET, moral leaders encourage their followers to engage in dyadic open communication to raise their ideas and suggestions, which fosters mutual trust (Hannah *et al*., 2024), encourages followers to reciprocate by going the extra mile to seek new ways to improve services, and by enhancing the quality of job tasks through extra effort in the form of service innovation (Xie *et al*., 2024). As a result, moral leaders' integrity, fairness and caring behaviour positively foster affective commitment, creativity and innovation among employees. Thus, we propose that ML positively influence SIB among healthcare employees.

H3a. ML is positively related to SIB.

Moral leaders have close, social exchange relationships with their subordinates (Costa *et al*., 2022) and these high-quality exchange relationships result from ethical leaders' openness, sincerity, and two-way communication strategies, which increase followers' trust and encourage them to reciprocate in ways that are mutually beneficial and less harmful (Qi *et al*., 2020; Grobler and Grobler, 2021). Moreover, when leaders exhibit positive social exchange and fairness in the form of resource distribution, policy development and information sharing, this acts of leaders lead to minimise the employee negative behaviours (Hussain and Khan, 2019; Alamir *et al*., 2019). Thus, we expect that employees are less likely to engage in counterproductive work behaviours when their leader exhibits integrity, fair treatment, and selflessness and promotes high-quality social exchange relations.

H3b. ML is negatively related to CWB.

## Mediation mechanism

Perceived supervisor support (PSS) is an employee’s perception concerning whether their leaders value their contributions and care about their wellbeing and daily activities and performance (Eisenberger *et al*., 2002). Because leaders act as agents of an organisation, they are responsible for instructing, evaluating performance, providing job-related resources (working space, equipment, growth opportunities), and also providing holistic support and care for their employees' welfare (Karakitapoğlu-Aygün *et al*., 2019; Yang *et al.*, 2020).

Prior studies reported that a leader’s support is positively linked with various employee outcomes, such as work wellness (Heyns *et al*., 2021), job satisfaction (Crucke *et al*., 2021) and employee engagement (Imam *et al*., 2023). According to Eisenberger *et al*. (2002), the development of PSS is a natural human tendency while working with a supervisor in a positive environment, including favourable policies and a supportive culture. Furthermore, employees would view favourable treatment as an indication of favour and support at the workplace, which boosts their PSS level.

PL may have a dual influence on subordinates' perceptions regarding a leader’s support (Franken *et al*., 2020). In some contexts, due to the leaders' strict behaviour to maintain discipline and rule of law, employees may perceive less democratic working environment and less support from leaders (Thoroughgood *et al*., 2018). In contrast, leaders' benevolent and moral behaviour provide individualised support in the form of wellbeing, care, mentorship, guidance, morality, honesty, justice and ethical conduct at the workplace (Kalliath *et al*., 2020) which may develop perceived support from supervisor. We propose that PSS is critical in cultivating a supportive working environment in the healthcare sector (Gordon, 2020) and may play a mediating role between PL and employee service behaviours, i.e. stimulate innovative ideas and lessen the CWBs. On this basis, we propose the following hypotheses:

H4a. PSS mediates the relationship between AL and SIB.

H4b. PSS mediates the relationship between BL and SIB.

H4c. PSS mediates the relationship between ML and SIB.

H4d. PSS mediates the relationship between AL and CWB.

H4e. PSS mediates the relationship between BL and CWB.

H4f. PSS mediates the relationship between ML and CWB.

## Moderation mechanism

PSM (public service motivation) is a person’s individual beliefs, values, and attitudes, including concern for the larger public interest and actions, that motivates the individual to go beyond personal and organisational interest (Perry and Wise, 1990). PSM is an individual’s prosocial motivation and driving force toward a public mission (Petersen, 2021; Ritz *et al*., 2020), which is influenced by various sociohistorical factors such as parental influence, religious affiliation, and personal character. Perry and Wise (1990) proposed that fostering PSM may contribute to community services such as education, healthcare, public-sector and social-service organisations. For example, healthcare personnel plays a significant role in hospitals, and their motivation and behaviour impact how well service operations run (Morin and Talbot, 2024).

**Figure 1:**
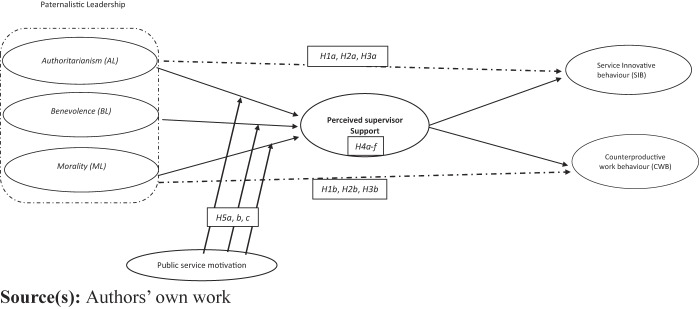
Research model

We propose PSM as a moderating variable to influence the relationship between PL dimensions and PSS (mediator) because PSM is aligned with a leader’s perceived support and leadership behaviour (benevolence) where leaders display concern for employees' personal and professional welfare. Furthermore, healthcare workers with high PSM are likely to respond positively because they value collective welfare and are intrinsically motivated to contribute to organisational and societal goals. PSM can work as an individual resource on which employees draw to develop their repertoire of organisational and social resources to serve public concerns (Luu, 2021). We also propose that PL dimensions may influence employees' reciprocity feelings to their outcomes depending on their perceived supervisor’s support. Individuals with a high level of PSM are self-motivated and may express better performance, as they perceive commitment to serve the community (Deng *et al*., 2021). This aligns with values from benevolent and moral leaders. Individuals with high empathy levels are likely to seek public benefits from their actions (Potipiroon and Faerman, 2020), and their actions tend to be unrelated to the norm of reciprocity, as they secure personal motivation from their deeds for the collective good. Individuals with prosocial behaviour, such as commitment to public values, compassion, and sacrifice to the community, may respond positively to benevolent/moral leaders' behaviours. Furthermore, these individuals prevent themselves from engaging in behaviours that may harm the organisation and its stakeholders. This study expects PSM to serve as an individual control for the impact of PL dimensions on perceived support from leaders. Hence, we propose the following hypotheses: (see Figure 1).

H5a. PSM positively moderates the relationship between AL and PSS

H5b. PSM negatively moderates the relationship between BL and PSS.

H5c. PSM negatively moderates the relationship between ML and PSS.

## Methods

### Sample and procedures

We gathered data from healthcare employees (doctors, nurses) working in hospitals in Pakistan. The units of analysis were physicians/doctors (house officer, senior house officer, medical officer, senior medical officer) and nurses (registered staff nurse), as doctors and nurses are two of the main organisational actors in the healthcare sector in the context of patient care.

We utilised snowball sampling to recruit participants working in big hospitals (hospitals with at least 100 full-time employees) located in four big cities (Karachi, Lahore, Rawalpindi, and Faisalabad) of Pakistan. We contacted the first hospital randomly, and with the hospital management permission, we disseminated survey questionnaire. We used snowball sampling because finding an initial pool of healthcare professionals (willing participants) was highly based on connections (Schlef *et al*., 2018). We intentionally selected participants based on their experiences working in hospitals and interacting with hospital managers (head doctors or nurses) (Alnjadat *et al*., 2024). Also, the initial pool of respondents was approached to participate in the research study through acquaintances in alumni groups (associations or groups of former students) of medical schools and healthcare professional/social groups.

A questionnaire survey was distributed via Qualtrics, an online platform, to healthcare professionals working in public, private, and semi-government hospitals. Our data collection via Qualtrics ensured anonymity for participants, and the research was conducted according to Swinburne University’s ethics guidelines. The survey link was accompanied by detailed information about the research study aims, guaranteed response anonymity and confidentially, and encouraged them to share the link with their colleagues and professional groups. After two weeks, a reminder email was sent to the initial pool of participants. The main challenge was securing an appropriate number of respondents, which was overcome by the initial pool of participants helping to relay the questionnaire link to their colleagues working in the same capacity. Also, the managers of the initial hospital introduced us to some other hospitals, which helped to enhance the response rate.

At the initial stage, we collected 618 responses over a period of ten weeks. To improve the quality of data, we screened the data by removing incomplete responses or entire cases (more than 10% missing observations of a response), pattern responses which follow a specific pattern (responses such as 1,1,1,2,2,2,3,3,3 or 1,2,3,4,5 or 1,1,1,1,1 or 5,5,5,5,5), outliers, random missing data and non-random missing data (Tsikriktsis, 2005). Furthermore, we applied various methods such as Mahalanobis distance, Cook’s distance, and leverage scores (Tabachnick *et al*., 2007) to remove outliers. After data screening, 319 valid responses out of 618 were obtained, which met the required minimum sample requirement for SEM using G*power (137 participants). Through this process, we also met the requirement of sample-to-item ratio (5:1) (Hair *et al*., 2010), with researchers (Hox and Maas, 2001; Wolf *et al*., 2013) suggesting that a minimum of 100 participants is required.

### Measures

We utilised validated scale items with a five-point Likert scale (1 = strongly disagree to 5 = strongly agree) to measure the constructs. We utilised the short version scale of paternalistic leadership developed by Cheng and Wang (2015). The sample items included “*Authoritarianism:* My team leader always has the last say in the meeting”, “*Benevolence:* My team leader ordinarily shows a kind concern for my comfort”, and “*Morality:* He/she is unselfish toward us”. For outcome variables, a nine-items scale to measure SIB (sample item: He/she is involved in creating new ideas for difficult issues) was used, developed by Scott and Bruce (1994). For CWB, three items (sample item: He/she intentionally arrives late for work regularly) were taken from Van Jaarsveld *et al.* (2010), and an additional two items (sample item: He/she intentionally slowed his service to a customer) were taken from Shao and Skarlicki (2014).

We utilised a 4-item short-version scale to measure PSM (Perry, 1997). The sample item is “Making a difference in society means more to me than my achievements”. We adopted validated scales for the measurement of PSS (sample item *“*My manager/supervisor cares for what I think”), developed by Jin and McDonald (2017). We used gender, education, working tenure with current supervisor, working tenure in the current organisation, and organisation type as control variables.

## Common method bias (CMB)

We utilised procedural and statistical treatments to mitigate the risk of CMB (Podsakoff *et al*., 2003). Following procedural treatments (Kock *et al*., 2021), we provided a research information cover letter describing instructions, voluntary participation, participants' anonymity, data being used for academic research purposes, and the freedom to quit participation, if respondents felt uncomfortable at any stage. In addition, we mixed the questionnaire items so that it is difficult to identify any construct name, so as to avoid any response pattern.

To address CMB concerns, the following statistical procedures were taken. First, the study used the Harman single-factor model test. With a KMO (Kaiser-Meyer-Olkin) score of 0.816, below the 50% cut-off value (Fornell and Larcker, 1981), this test’s results showed that a single component without any rotation adequately explained 19.893% of the variance, indicating insufficient risk of data bias. Second, we used confirmatory factor analysis (CFA) to apply a common latent factor strategy. We discovered that the constrained and unconstrained models were the same, and the test did not reveal any bias because we loaded all of the items into a single factor known as the “common latent factor”, as suggested by Podsakoff *et al*. (2003). Third, we measured variance inflation factors (VIF) for each independent construct (VIF range values 1.066–1.631) and found it within a cut-off value of 3.3 (Kock, 2015). Moreover, tolerance was calculated for each independent variable, and the range of tolerance values was far higher (0.3) than the critical value of 0.1. These results reflected no serious issues regarding multicollinearity and CMB (Kock *et al*., 2021).

### Data analysis

The data analysis strategy included validity and reliability of the scale, model fit, and comparison of different measurement models. We calculated the direct, indirect, and total impacts of the correlational paths to analyse the mediation paths. In the next step, mediation examined path coefficients and Monte Carlo simulation results for mediation correlations. We also calculated interaction terms with slope graphs representing the interactional impacts on the correlations to measure the moderating effects.

## Results and findings

### Preliminary analyses

The demographic results indicate that more females (57.1%) participated in the study than males (42.9%). Most participants were aged 26–35 (48.9%) and 18–25 (41.1%).

We conducted a pilot study (50 responses) before the main study which helped us make minor modifications in the phrasing of items for clarity purposes. The factor loadings of items in the pilot study ranged from 0.544 to 0.823 which are within the recommended range. We cleaned data for the main study by handling missing values and outliers with the Mahalanobis distance and Cook’s distance tests (Desimone *et al*., 2015). To determine data normality, we examined factor loadings, skewness and kurtosis coefficients (fall in the range ±1.96 and ± 3, respectively), mean and standard deviations of each item for the main study. On this basis, it could be assumed that the data of our study were normally distributed to meet the basic SEM assumption. Moreover, multicollinearity did not appear as a serious issue in this model and the correlation values among independent variables (AL, BL, ML, PSS) were found to be under the limit (high correlation value 0.8) (Hair *et al*., 2010) (see Table 1).

**Table 1:** Correlation

		1	2	3	4	5	6	7	8	9	10	11	12	13
1	Age													
2	Occupational role	0.210^**^												
3	Gender	−0.035	0.310^**^											
4	Tenure in organisation	0.562^**^	0.242^**^	−0.030										
5	Tenure with current leader	0.307^**^	0.159^**^	0.045	0.475^**^									
6	Organisation type	−0.105	−0.190^**^	−0.044	0.019	0.102								
7	AL	0.063	0.102	0.022	−0.075	−0.024	−0.066							
8	BL	−0.134^*^	−0.095	−0.050	−0.075	−0.011	0.181^**^	−0.172^**^						
9	ML	−0.119^*^	−0.178^**^	−0.035	−0.084	−0.002	0.133^*^	−0.283^**^	0.387^**^					
10	PSS	−0.124^*^	−0.095	0.092	−0.103	−0.003	0.124^*^	−0.306^**^	0.317^**^	0.452^**^				
11	PSM	0.059	−0.032	0.009	0.091	0.031	0.028	−0.010	0.174^**^	0.172^**^	0.097			
12	SIB	0.071	−0.007	−0.045	0.046	0.008	−0.033	−0.253^**^	0.106	0.219^**^	0.296^**^	0.304^**^		
13	CWB	0.278^**^	0.165^**^	0.138^*^	0.198^**^	0.053	−0.191^**^	−0.016	−0.210^**^	0.036	0.007	0.088	0.188^**^	

**Note(s):** (PSS: AL: authoritarian leadership, BL: benevolent leadership, ML: moral leadership, PSS: perceived supervisor support, PSM: public service motivation, SIB: Service Innovative Behaviour, CWB: counterproductive working behaviour, 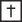 *p* < 0.1, *p < 0.05, **p < 0.01, ***<0.001)

**Source(s):** Authors’ own work

### Validity and reliability of the scale (main study)

We measured composite reliability (CR), average variance extracted (AVE) and maximum shared squared variance (MSV) to examine the validity and reliability of the constructs (Hu and Bentler, 1999). Further, discriminant validity is considered to be attained when AVE is greater than MSV, whereas CR and AVE measure the convergent validity of the scale through confirmatory factor analysis (CFA).

Table 2 shows that the CR and AVE values, most of the latent variables, exceeded the recommended cut-off values of 0.7 and 0.5 (Hair *et al*., 2017). However, CR values of some latent variables such as AL (0.669), BL (0.686) and PSM (0.662) were a little below 0.7, which is in an acceptable range of 0.6 for CR recommended by Fornell and Larcker (1981) and consistent with recent research (Fenitra *et al*., 2023; Luna-Arocas and Danvila-del-Valle, 2021; Xiao and Lam, 2020). Similarly, some of the constructs such as PSS, SIB and PSM range AVE values were between 0.45–0.5, which is slightly lower 0.5, but are in an acceptable value of 0.4 as suggested by Fornell and Larcker (1981). Furthermore, the Cronbach alpha (α) of latent variables ranged between 0.86-0.658, surpassing the minimum acceptable limit of 0.6 (Fornell and Larcker, 1981; Taber, 2018).

**Table 2:** Validity analysis

	CR	AVE	MSV	MaxR(H)	SIB	PSS	CWB	ML	PSM	AL	BL
SIB	0.843	0.474	0.222	0.848	*0.689*						
PSS	0.778	0.469	0.237	0.789	0.336***	*0.685*					
CWB	0.754	0.534	0.237	0.758	0.206**	−0.001	*0.659*				
ML	0.795	0.567	0.266	0.811	0.215**	0.486***	0.038	*0.753*			
PSM	0.674	0.515	0.167	0.726	0.350***	0.144†	0.142†	0.247**	*0.717*		
AL	0.718	0.447	0.190	0.618	−0.357***	−0.436***	−0.026	−0.408***	0.011	*0.669*	
BL	0.685	0.532	0.266	0.777	0.143*	0.428***	−0.300***	0.515***	0.212**	−0.315**	*0.729*

**Source(s):** Authors’ own work

The current model met the validity and reliability standards. We compared the square root of AVE with the correlation of latent variables (Fornell and Larcker, 1981) to assess discriminant validity through heterotrait-monotrait (HTMT). The HTMT ratio of correlations among latent variables where the squared root value of AVE mentioned in the diagonal is greater than its correlation values presented horizontally and vertically (Table 2).

### Measurement models

Common indices (CMIN/DF, GFI, CFI, TLI, RMEA and SRMR) are used to measure the fitness of the measurement model in social science research. Table 3 summarises a decent model fit of our proposed model (X^2^/df = 1.832 < 2, TLI = 0.905, CFI = 0.920, RMSEA = 0.051, SRMR = 0.051) compared to alternative models. For example, the seven-factor model presented better fits than the fits of the six-factor model by combining authoritarian (AL) and benevolent leadership (BL). Similarly, the six-factor model showed better fits than the five-factor model fits (AL, BL and ML combined).

**Table 3:** Comparison of measurement models

Model	Chi-square	CMIN/DF	CFI	TLI	RMSEA	SRMR	PClose
7 Factor model	496.510	1.832	0.920	0.905	0.051	0.051	0.386
6 Factor model, AL, and BL combined	538.859	2.12	0.887	0.866	0.059	0.061	0.014
5 Factor model, AL, BL, and ML combined	609.449	2.344	0.861	0.840	0.065	0.065	0.000
4 Factor model, AL, BL, ML and PSS combined	761.550	2.874	0.803	0.777	0.077	0.080	0.000
3 Factor model AL, BL, ML, PSS, and PSM combined	937.994	3.487	0.734	0.704	0.088	0.089	0.000

**Source(s):** Authors’ own work

### Hypotheses testing (main study)

Table 4 exhibits that AL and ML demonstrated a non-significant direct relationship with both outcome variables. BL was found, however, to be positively significant to SIB (β = 0.976, *p* = 0.001 and negatively significant with CWB (β = −1.800, *p* = 0.001), providing support for hypotheses H1b and H2b. Direct relationships from PL dimensions to the mediating variable (PSS) were correlated significantly. Further, PSS correlated significantly with SIB and CWB in direct association.

**Table 4:** Hypotheses testing

Hypotheses	Direct paths			Estimate	S.E.	C.R.	P
H1a	AL	→	SIB	−0.070	0.046	−1.527	0.127
H1b	AL	→	CWB	0.070	0.071	0.987	0.324
H2a	BL	→	SIB	0.976	0.266	3.667	***
H2b	BL	→	CWB	−1.800	0.467	−3.853	***
H3a	ML	→	SIB	−0.009	0.053	−0.176	0.860
H3b	ML	→	CWB	0.008	0.085	0.095	0.924

Hypothesis	Indirect path	Unstandardized estimate	Lower limit	Upper limit	*p*-value	Standardized estimate	Result (mediation)
H4a	AL → PSS → SIB	−0.095	−1.312	−0.016	0.021	−0.188*	Full Mediation
H4b	BL → PSS → SIB	0.697	0.028	3.949	0.027	0.477*	Partial Mediation
H4c	ML → PSS → SIB	0.128	0.034	0.553	0.020	0.200*	Partial Mediation
H4d	AL → PSS → CWB	−0.105	−1.434	−0.005	0.066	−0.144 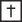	Full Mediation
H4e	BL → PSS → CWB	0.771	0.008	4.873	0.072	−0.367 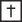	Full Mediation
H4f	ML → PSS → CWB	−0.141	−0.019	−0.678	0.054	−0.154 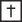	Full Mediation

**Source(s):** Authors’ own work

### Mediation

**Figure 2:**
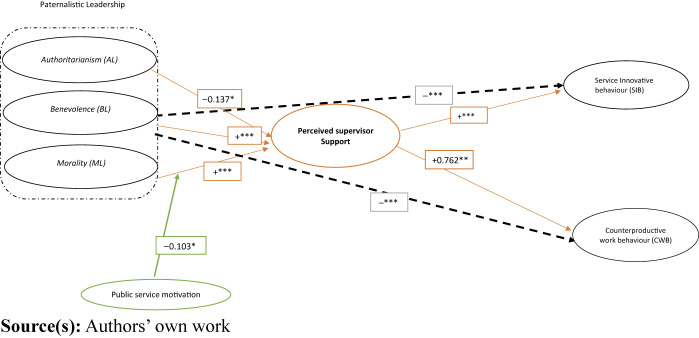
Research Model with results

Following the Sobel test (Sobel, 1982) and bootstrapping approach (Preacher *et al*., 2010), the indirect effect of PL dimensions to SIB and CWB via PSS shows full mediation except for BL and ML to SIB, which exhibits partial mediation (Table 4). These results demonstrated a 95% confidence interval with no straddling zero. Moreover, these results support the indirect influence of PSS to promote SIB and reduce CWB (H4a-H4f) (see Figure 2).

### Moderation

**Figure 3:**
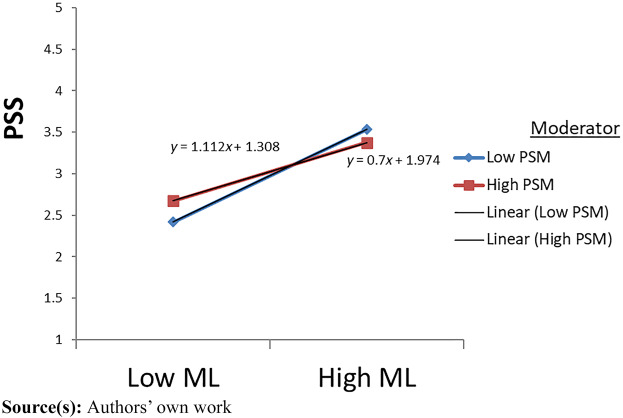
Moderating effect of PSM on ML and PSS

We estimated interaction effects by utilising interaction term coefficients and drawing slopes. We posited that PSM would moderate between PL dimensions (AL, BL, and ML) and the mediating variable (PSS). The interaction term of ML X PSM (H5c) in predicting perceived supervisor support was negative (β = −0.103, *p* < 0.05), and the rest of the two interaction terms (AL X PSM, BL X PSM) (H5a and H5b) were non-significant (see Table 5) (see Figure 3).

**Table 5:** Moderation

Hypothesis	Independent variable (IV)	Dependent variable (DV)	Regression weight on DV			Result (moderation)
			IV	Mod	IAT	
H5a	Authoritative Leadership	Perceived Supervisory Support	−0.311***	0.096	0.049	No moderation
H5b	Benevolent Leadership	Perceived Supervisory Support	0.383***	0.005	−0.076	No moderation
H5c	Moral Leadership	Perceived Supervisory Support	0.453***	0.024	−0.103*	Moderation

**Note(s):** *IAT: Interaction term, IV: Independent variable, DV: Dependent variable, Mod: Moderator

**Source(s):** Authors’ own work

## Discussion

The findings from the hypotheses testing confirmed that employees' SIB and CWB are important in the context of PL style in the healthcare sector. Results confirmed that the benevolence of paternalistic leaders enhances employee service innovative behaviour (H2a) and mitigates counterproductive working behaviour (H2b). These results are consistent with prior studies (Nazir *et al*., 2020; Yao and Hao, 2023; Zhang *et al*., 2023; Rui and Xinqi, 2020) where the benevolent behaviour of the leader has been found to enhance SIB in various service sectors and to reduce employees' counterproductive working behaviour.

In contrast, the direct associations of AL and BL to employee outcomes (SIB and CWB) were not significant (H1a, H1b, H3a and H3b not supported). There could be various reasons behind the non-significant results, such as individual factors, personality traits of followers, organisational environment, cultural values of a specific context, and the need for mediating variables. For instance, a possible reason for the non-significant results between PL dimensions (AL and ML) and outcome variables (SIB, CWB) would be that healthcare workers do not fully depend on instructions to do a task. They have clear standard operating procedures, job descriptions and required roles to perform, while also needing to use their professional judgement in individual cases. Also, there is a possibility that employees only perform required job duties, and do not undertake extra role behaviour and unethical work behaviours. Moreover, cultural factors cannot be ignored. In this regard, Pakistan is a collectivist and high-power distance society, in which authoritarianism due to strict discipline may influence employees to fulfil in-role behaviour and avoid negative behaviour.

Our findings also revealed that PSS played an important role in shaping positive and negative working behaviour. For instance, PSS fully mediated the relationship between outcome variables (SIB and CWB). Similarly, PSS fully mediated the associations between BL and CWB. This provides a contribution to existing literature, showing that PSS can play a significant role in mitigating negative behaviours among employees. Our findings are consistent with prior studies (Chan, 2017; Chen *et al*., 2014; Lee *et al*., 2019) which found that PL (BL and ML) behaviour was associated with PSS, which enhances followers' positive outcomes. Moreover, SET explains the association between PL behaviour and followers' outcomes through PSS. This suggests that PSS is a significant antecedent influencing followers' outcomes.

Results further indicated the moderating effect of PSM (public service motivation) on the relationship between PL dimensions and PSS (see *H5 (a-c)*. Our findings revealed that PSM was negative and significant to moderate the relationship between ML and PSS which is consistent with recent research (Luu, 2021). These findings show that individuals with high PSM may not necessarily require moral treatment from their leaders to foster perceived supervisory support. These individuals are self-motivated to serve the community and may not be influenced by the PL style. Additionally, this finding indicates that healthcare employees may draw upon PSM as a proximal and alternate resource to perform job duties and may rely less on support from leaders. However, when individuals lack PSM as a resource, they are more reliant on the supervisor’s support as an alternative resource for their job performance. In this way, our study advances the PL literature by examining PSM as a moderator, whereas prior PL research has focused on contextual or individual moderators in relation to employee behaviours.

## Theoretical contribution

This study makes several theoretical contributions. First, this study extends the PL literature by demonstrating that PL style can simultaneously influence both positive (SIB) and negative (CWB) working behaviours simultaneously at an individual level in the healthcare industry. While some studies have focused on PL and innovative work behaviour (Papa *et al*., 2020; Nazir *et al*., 2020; Zhang *et al*., 2022), only limited scholarly attention has investigated SIB and CWB behaviours simultaneously in a single study (Yue *et al*., 2024). With increasing demands from patients on healthcare professionals, our findings indicated that SIB and CWB are aligned with PL.

Second, this study confirms the mediating role of PSS (full and partial mediation) between PL dimensions and service behaviours. Our study varies from existing PL models by furthering our understanding of PL investigation through PSS meditation (*H4a-H4f*) for SIB and CWB. Existing studies have investigated the effects of PL dimensions with different mediating variables such as self-efficacy (Wang *et al*., 2022), job embeddedness (Khorakian *et al*., 2021), team performance (Huang and Lin, 2021) and work-family conflict (He *et al*., 2021). Based on leadership concepts, the results of the present study highlight the role of perceived supervisory support in stimulating SIB and mitigating CWB among employees.

Third, we found the moderating role of PSM diminished the positive relationships between moral leadership and PSS. Furthermore, by identifying PSM as a moderating factor in the relationship between PL dimensions and mediator (PSS), our study responds to the call for further investigations into boundary conditions behind the effects of PL (Petersen, 2021; Fernandes *et al*., 2022). Prior PL studies have examined different moderating variables, such as cross-cultural adaptability (He *et al*., 2021), voice behaviour (Jia *et al*., 2020), and flexible role identity (Luu and Djurkovic, 2019). Our research findings contribute to the PL literature by extending PSM beyond the public administration area to a sector (healthcare) where PSM can be a crucial force driving employees to serve patients and the community.

Fourth, our findings further strengthen the implications of SET and enhance the explanatory power of the social exchange process by identifying SIB and CWB as positive and negative behaviours formed among employees based on the social exchange relationships that they forge with the leader exhibiting different PL dimensions. Prior studies used SET to draw empirical connections between PL dimensions and various job outcomes such as creativity (Khorakian *et al*., 2021), burnout (Chang *et al*., 2019), and productive energy (Grobler and Grobler, 2021) in the tourism, manufacturing and education industries. This study extends SET by integrating a contingency perspective with SET by identifying the boundary condition (that is, PSM) that can influence how employees respond to different leadership dimensions.

### Managerial implications

This research provides several implications for healthcare managers. First, our study suggests that benevolent leadership is positively associated with SIB and negatively with CWBs among healthcare workers. Because clinical jobs are high pressure, authoritative leadership should be avoided when promoting SIB and reducing CWB. Our findings suggest that healthcare managers should practice benevolent leadership styles by caring about healthcare employees' work conditions and their needs for performance and career growth as well as providing support for fulfilling their workload. Healthcare managers should also balance the three dimensions of PL by using less authoritarianism in everyday activities and by using authoritarianism only in specific situations such as in cases of emergency or the strict application of some medical interventions. Healthcare managers should not overuse benevolent leadership since this may promote employees' dependency on manager care or stifle critical feedback from managers. Overall, managers need to balance authoritarianism, benevolence, and morality so that authoritarian behaviour does not dominate the overall leadership style or vice versa (Pizzolitto *et al*., 2022). In a dynamic environment, the healthcare sector should benefit from the innovative behaviours of employees, as our findings suggest that the benevolence dimension of PL is directly associated with SIB. Further, benevolence mitigates the effects of negative working behaviours (CWB).

Second, this study provides evidence that perceived supervisor support tends to intervene positively in the relationship between leaders and employee outcomes. There is a need to develop a high-quality support perception about managers and organisations among doctors and nurses because they have stressful job duties, such as diagnosis, medical treatment, surgical treatment and post-operative care. This support perception needs to be earned through the actions of managers and organisations.

Third, this study suggests that PSM is a specific motivation force among healthcare workers. Managers should understand the critical role of PSM as an internalised motivation factor and should integrate public service values into their management system.

Lastly, in the light of study findings, the government and boards of directors should arrange training programs for hospital managers at the national level. Policymakers should advocate for integrating leadership development into national healthcare strategies, ensuring that training focuses on skills such as paternalistic leadership and using a balance of the three dimensions. This will be beneficial not only for the current managers but also for the succession planning of healthcare managers and CEOs in hospitals, especially public hospitals. It may shape service behaviour positively among healthcare employees, which meets societal expectations of healthcare service quality.

## Limitations and future research directions

There are some limitations of this study. First, the survey data was collected from Pakistani hospitals, meaning that these results cannot be generalised to other sectors or to other countries. Future research should replicate this model in different service sectors, such as education, hospitality, and public sector institutions, as well as in various countries.

Second, a cross-sectional design was used to gather the data. As such, it is hard to measure causal inference and infer causal associations. Future research should adopt a longitudinal cross-lagged approach or experimental designs to infer causality among variables.

Third, we minimised the risk of CMB (Podsakoff *et al*., 2003) by performing different procedural and statistical treatments such as Harman’s single factor test, VIF, tolerance and expanded sample test of more than 250 participants in SEM (Hu and Bentler, 1999). To further reduce such biases or errors, future studies should collect separate datasets through split survey administration, for example, employee-rated data related to leadership and leader-rated data related to employee outcomes.

Fourth, this research model considered two extra-role service behaviours: SIB and CWB. Future studies should assess different employee outcomes (turnover intentions and employee burnout) and mediation mechanisms (organisational trust and commitment).

Fifth, this study focused on PSM as a moderating variable between the associations of PL and a mediating variable. Future studies should consider other moderating factors such as workplace stress and emotional intelligence. Furthermore, the findings related to PSM moderation are limited to Pakistani healthcare context and cannot be generalised to other sectors or national contexts.

## Conclusion

This study aimed to develop an integrated understanding of PL, its antecedents, outcomes, mediation factor, and moderation factor in the context of the Pakistani hospital system. In this way, the study contributed to the PL literature by examining PL with positive and negative followers' outcomes collectively through a mediation (PSS) and moderation (PSM) mechanism. Our findings supported key results from prior studies, while also adding new insights to the PL literature by proposing a new research model. The study findings highlighted the significance of PL dimensions in directly and indirectly predicting employee behaviours. Furthermore, the results revealed the role of support from the supervisor in a mediating mechanism that extends existing knowledge underlying the support from leaders to shape followers' outcomes. Additionally, this study highlighted the moderating role of PSM in the relationship between PL dimensions and PSS. This study also contributed to enhancing the application of social exchange theory. Overall, this study has demonstrated the value of PL, while showing that PL remains an emerging research area in leadership studies requiring further systematic investigations in diverse organisational and national contexts.
